# Supplementary material for: Multi‐dataset identification of innovative feature genes and molecular mechanisms in keratoconus
Source: J Cell Mol Med. 2024 Sep 19;28(18):e70079. doi: 10.1111/jcmm.70079 (PMC11412914; doi:10.1111/jcmm.70079)
Supplement: Supplementary file 2 — Material S2. [file JCMM-28-e70079-s002.zip › 7.GSEA analysis/5.RNF217-AS1/KEGG_PROPANOATE_METABOLISM.html]

Details for gene set KEGG\_PROPANOATE\_METABOLISM[GSEA]

|  || Dataset | RNF217-AS1.group.cls#h\_versus\_l |
| Phenotype | group.cls#h\_versus\_l |
| Upregulated in class | l |
| GeneSet | KEGG\_PROPANOATE\_METABOLISM |
| Enrichment Score (ES) | -0.38708845 |
| Normalized Enrichment Score (NES) | -1.2563854 |
| Nominal p-value | 0.24248497 |
| FDR q-value | 0.4326594 |
| FWER p-Value | 0.968 |
Table: GSEA Results Summary

  

Fig 1: Enrichment plot: KEGG\_PROPANOATE\_METABOLISM      
 Profile of the Running ES Score & Positions of GeneSet Members on the Rank Ordered List

  

| SYMBOL | TITLE | RANK IN GENE LIST | RANK METRIC SCORE | RUNNING ES | CORE ENRICHMENT || 1 | SUCLG2 | na | 4742 | 0.226 | -0.0588 | No |
| 2 | LDHAL6A | na | 6613 | 0.164 | -0.0560 | No |
| 3 | ABAT | na | 7227 | 0.146 | -0.0233 | No |
| 4 | ACADM | na | 15052 | 0.018 | -0.2424 | No |
| 5 | HIBCH | na | 15665 | 0.012 | -0.2559 | No |
| 6 | LDHA | na | 16042 | 0.008 | -0.2639 | No |
| 7 | LDHB | na | 16493 | 0.004 | -0.2756 | No |
| 8 | ACSS2 | na | 16840 | 0.000 | -0.2855 | No |
| 9 | ACAT2 | na | 17362 | -0.005 | -0.2987 | No |
| 10 | ACACA | na | 18261 | -0.013 | -0.3200 | No |
| 11 | SUCLG1 | na | 18904 | -0.019 | -0.3319 | No |
| 12 | ALDH3A2 | na | 20121 | -0.031 | -0.3562 | No |
| 13 | HADHA | na | 20646 | -0.036 | -0.3588 | No |
| 14 | ALDH6A1 | na | 20921 | -0.039 | -0.3532 | No |
| 15 | ECHS1 | na | 20997 | -0.040 | -0.3416 | No |
| 16 | ACAT1 | na | 22576 | -0.057 | -0.3675 | Yes |
| 17 | MCEE | na | 22902 | -0.061 | -0.3559 | Yes |
| 18 | ACSS1 | na | 22928 | -0.061 | -0.3357 | Yes |
| 19 | SUCLA2 | na | 23100 | -0.063 | -0.3189 | Yes |
| 20 | ALDH9A1 | na | 23254 | -0.065 | -0.3010 | Yes |
| 21 | ALDH7A1 | na | 25259 | -0.095 | -0.3261 | Yes |
| 22 | ACSS3 | na | 25383 | -0.097 | -0.2963 | Yes |
| 23 | PCCB | na | 25399 | -0.097 | -0.2633 | Yes |
| 24 | LDHC | na | 25553 | -0.099 | -0.2335 | Yes |
| 25 | LDHAL6B | na | 26559 | -0.117 | -0.2221 | Yes |
| 26 | EHHADH | na | 27336 | -0.133 | -0.1986 | Yes |
| 27 | PCCA | na | 27841 | -0.145 | -0.1633 | Yes |
| 28 | ACACB | na | 27956 | -0.147 | -0.1158 | Yes |
| 29 | ALDH1B1 | na | 28642 | -0.166 | -0.0782 | Yes |
| 30 | MLYCD | na | 32343 | -0.312 | -0.0772 | Yes |
| 31 | ALDH2 | na | 33631 | -0.423 | 0.0316 | Yes |
Table: GSEA details [plain text format]

  

Fig 2: KEGG\_PROPANOATE\_METABOLISM      
 Blue-Pink O' Gram in the Space of the Analyzed GeneSet

  

Fig 3: KEGG\_PROPANOATE\_METABOLISM: Random ES distribution      
 Gene set null distribution of ES for **KEGG\_PROPANOATE\_METABOLISM**
